# Dual-molecular barcode sequencing detects rare variants in tumor and cell free DNA in plasma

**DOI:** 10.1038/s41598-020-60361-3

**Published:** 2020-02-25

**Authors:** Yosuke Hirotsu, Sotaro Otake, Hiroshi Ohyama, Kenji Amemiya, Rumi Higuchi, Toshio Oyama, Hitoshi Mochizuki, Taichiro Goto, Masao Omata

**Affiliations:** 1 Genome Analysis Center, 1-1-1 Fujimi, Kofu, Yamanashi, Japan; 2 Lung Cancer and Respiratory Disease Center, 1-1-1 Fujimi, Kofu, Yamanashi, Japan; 3 Department of Gastroenterology, Yamanashi Central Hospital, 1-1-1 Fujimi, Kofu, Yamanashi, Japan; 4 Department of Gastroenterology, Chiba University, Graduate School of Medicine, Inohana, Chuo-ku, Chiba, Japan; 5 Department of Pathology, Yamanashi Central Hospital, 1-1-1 Fujimi, Kofu, Yamanashi, Japan; 6 The University of Tokyo, 7-3-1 Hongo, Bunkyo-ku, Tokyo, Japan

**Keywords:** Diagnostic markers, Tumour biomarkers

## Abstract

Conventional next generation sequencing analysis has provided important insights into cancer genetics. However, the detection of rare (low allele fraction) variants remains difficult because of the error-prone nucleotide changes derived from sequencing/PCR errors. To eliminate the false-positive variants and detect genuine rare variants, sequencing technology combined with molecular barcodes will be useful. Here, we used the newly developed dual-molecular barcode technology (Ion AmpliSeq HD) to analyze somatic mutations in 24 samples (12 tumor tissues and 12 plasma) from 12 patients with biliary-pancreatic and non-small cell lung cancers. We compared the results between next generation sequencing analysis with or without molecular barcode technologies. The variant allele fraction (VAF) between non-molecular barcode and molecular barcode sequencing was correlated in plasma DNA (R^2^ = 0.956) and tumor (R^2^ = 0.935). Both methods successfully detected high VAF mutations, however, rare variants were only identified by molecular barcode sequencing and not by non-molecular barcode sequencing. Some of these rare variants in tumors were annotated as pathogenic, and therefore subclonal driver mutations could be observed. Furthermore, the very low VAF down to 0.17% were identified in cell free DNA in plasma. These results demonstrate that the dual molecular barcode sequencing technologies can sensitively detect rare somatic mutations, and will be important in the investigation of the clonal and subclonal architectures of tumor heterogeneity.

## Introduction

Cancer acquires somatic mutations during the evolution of a tumor. Subclonal mutants are considered to be associated with drug resistance in various cancers, including non-small cell lung, breast and colorectal cancers^1,2^. Cell free DNA (cfDNA) in plasma contains a small fraction of tumor DNA with tumor-derived mutations, which is called circulating tumor DNA. Plasma cfDNA is useful for monitoring tumor recurrence, estimating treatment effects and identifying drug-resistant mutations^3^. However, only low levels of mutated alleles are present in the overall cfDNA circulating in blood. Therefore, the development of highly sensitive methods to detect rare variants is required.

Various sensitive and accurate methods have been developed for the detection and quantification of mutated alleles in low abundance among high amounts of the wild-type allele^4^. These methods are important for medical oncology, cancer research, infectious disease and microbial studies. To investigate the tumor heterogeneity and cfDNA in liquid biopsy, highly sensitive assays are necessary for detecting somatic mutations with low variant allele fraction (VAF). Droplet digital PCR, chip-based digital PCR and beads, emulsion, amplification, magnetics and flow cytometry (BEAMing) assays can sensitively detect rare mutations present at 0.1% VAF^4^. Digital PCR and BEAMing have been applied for well-known pathogenic variants and detect several types of variants simultaneously; however, these may not be suitable for targeting a large number of genomic regions.

Next generation sequencing (NGS) technologies can provide high-scale sequencing data of genomic regions of interest. Conventional NGS analysis yields a large number of sequence reads and determines genetic changes, including single nucleotide variants, insertions, deletions, fusion, copy number variants and structural variants. High-depth sequencing data are especially important for detecting somatic mutations present at low frequency. PCR is usually conducted to amplify DNA and enrich adaptor-ligated fragments for sequencing. However, these procedures are associated with intrinsic errors (*i.e*. PCR and sequencing errors). Therefore, conventional NGS analysis has limitations in detecting somatic mutations with 2–5% VAF^5^. If we used conventional NGS assay, it will be difficult to detected true-positive somatic mutations with VAF below 1–2% due to PCR and sequencing artefacts^6,7^. We need to consider new strategies to detected true-positive low VAF mutations.

Novel assays have been developed to detect rare variants using NGS technologies combined with molecular barcode (MB) technologies^8^. This technology is commonly based on the approach that tagging individual DNA fragment with short random oligonucleotides called as unique molecular identifiers (UMI) or unique molecular tag (UMT)^8–10^. The molecular barcode discriminates original DNA, clusters the identical barcoded-reads and excludes error-prone nucleotide changes. Based on these technologies, Kinde *et al*. developed the approach of massively parallel sequencing using MB, which is called as Safe-Sequencing System (Safe-SeqS)^8^. Safe-SeqS increased the accuracy and sensitivity and easily be used to identify rare mutants in a population of DNA templates. Newman *et al*. reported that cancer personalized profiling by deep sequencing (CAPP-Seq) combined with integrated digital error suppression could eliminate the background artifacts and improved sensitivity and specificity^9^. Molecular barcode sequencing with deep coverage is useful for applying to detect low VAF mutations in cell free DNA in blood^3,11,12^. Several commercially available kits using MB technologies are available. SureSelect XT HS and HaloPlex (Agilent) are hybridize-capture-based methods and QIAseq Targeted Panel (Qiagen) is an amplicon-based method. An alternative amplicon-based method called IonAmpliSeq HD was recently launched Thermo Fisher Scientific.

Here we designed a Non-MB-based (Ion AmpliSeq) and MB-based panel (Ion AmpliSeq HD) targeting biliary-pancreatic and non-small cell lung cancers for analysis of somatic mutations in tumor tissues and plasma cfDNA. We compared the variant results between Non-MB and MB sequencing.

## Materials and Methods

### Patients and sample preparation

This study included 12 patients diagnosed with cholangiocarcinoma (n = 2; case #1 and #3), gallbladder cancer (n = 1; case #2), pancreatic cancer (n = 1; case #4), or non-small cell lung cancer (n = 8; case #5–12) at our institute. Informed consent was obtained from all patients. This study was approved by the Institutional Review Board of clinical research and genome research committee at Yamanashi Central Hospital (G-2018-1) and complied with Declaration of Helsinki principles. All peripheral blood samples were taken before biopsy, operation and cytology test from patients who did not received any treatment. Peripheral blood samples were collected in EDTA-2Na containing tube (Terumo, Tokyo, Japan) and centrifuged within 2 hours after collecting blood at 820 × *g* at 25 °C for 10 min, and buffy coats were isolated. Supernatants were centrifuged at 20,000 × *g* at 25 °C for 10 min to remove debris. Buffy coat and plasma were stored at −80 °C until DNA extraction. Tumor tissues were obtained by surgically resected tissues (n = 9; case #3 and #5–12), biopsies (n = 2; case #1 and #2) and cytology (n = 1; case #4). All tumor tissues and biopsy samples were fixed with 10% neutral buffered formalin and paraffin-embedded. Cytological specimens were fixed with 95% ethanol and stained with Papanicolau staining as previously described^13^.

For serial dilution analysis, we used EGFR Multiplex cfDNA Reference Standard Set (Horizon Discovery, Cambridge, UK) harboring engineered mutations. The mixtures represented 0.1%, 0.25%, 0.5%, 1%, 2.5% and 5% VAF range. The total number of DNA concentration was kept in constant (20 ng/µl).

### Buffy coat and plasma DNA extraction

Buffy coat DNA extraction was performed using the QIAamp DNA Blood Mini QIAcube Kit (Qiagen, Hilden, Germany) with the QIAcube (Qiagen) as previously described^14,15^. Briefly, 200 μL of buffy coat was incubated with Protease K and buffer AL. Genomic DNA was bound to the column, wash with Buffer AW1 and AW2, and eluted with Buffer AE. The concentration of DNA was determined using the NanoDrop 2000 spectrophotometer (Thermo Fisher Scientific, Waltham, MA, USA).

Plasma DNA was extracted using the MagMAX Cell-Free DNA isolation kit (Thermo Fisher Scientific) with KingFisher Duo Prime (Thermo Fisher Scientific) as previously described^16^. Briefly, 2–4 mL of plasma was mixed with Lysis/binding solution and magnetic beads. Beads were washed with Wash solution and 80% ethanol. Plasma DNA was eluted with 50 μL of Elution Buffer. The plasma DNA concentration was determined with the Qubit dsDNA HS Assay Kit and Qubit 3.0 Fluorometer (Thermo Fisher Scientific) in accordance with the manufacturer’s instructions.

### Laser capture microdissection and DNA extraction from FFPE and cytological specimen

Serial sections 10-μm-thick were prepared from FFPE tissues of surgical and biopsy specimens using Arcuturus PEN Membrane Glass Slides (Thermo Fisher Scientific)^17^. The sections were then deparaffinized and stained with hematoxylin-eosin. All slides were reviewed by a pathologist (T.O.) and cytotechnologist (K.A.) to check cellular content and characteristics as previously described^13^ (Supplemental Table 1). Laser-capture microdissection was performed using an Arcturus XT laser microdissection system (Thermo Fisher Scientific).

To obtain archival cytological specimen, the glass slides was soaked immersed in xylene to remove the cover glass. Using a razor blade, we directly scraped tumor cells from the entire slide. Tumor cells were collected into the sterile tube. DNA from surgical, biopsy specimens and cytological specimen extracted with the GeneRead DNA FFPE Kit (Qiagen, Hilden, Germany) in accordance with the manufacturer’s instructions. FFPE DNA was treated with uracil DNA glycosylase within the kit. To assess the quality and concentration of FFPE DNA, we used the TaqMan RNase P Detection Reagents Kit and the FFPE DNA QC Assay v2 on a ViiA 7 Real-Time PCR System (Thermo Fisher Scientific) as previously described^13^.

### Selecting genes and primer design

We made four in-house panels targeting biliary-pancreatic- or lung cancer-associated genes for Non-MB and MB sequencing. The Ion AmpliSeq primer set (Non-MB technology) and Ion AmpliSeq HD primer set (MB technology) were designed on Ion AmpliSeq Designer (Thermo Fisher Scientific). Amplicon length was designed to be short, because FFPE DNA is degraded during the fixation with formalin. Similarly, plsma DNA is fragmented to an approximate length of ~170 bp in blood circulation^18,19^. We designed the median size of PCR amplicons of the Non-MB and MB biliary-pancreatic panels as 112 bp (range: 61–137) and 118 bp (range: 72–133), respectively. The median sizes of amplicons of the Non-MB and MB lung cancer panels were 113 bp (range: 60–140 bp) and 117 bp (range: 75–135 bp), respectively.

The Non-MB-based biliary-pancreatic panel targeting 60 genes including whole exons and hotspots contained 2,820 primer pairs and spanned 280.22 kb (Table 1). To achieve high-depth data by MB sequencing analysis, genes and hotspot regions of interest were selected. The MB-based biliary-pancreatic panel targeting 21 genes contained 67 primer pairs and spanned 7.22 kb. The Non-MB-based lung cancer panel was used as previously described^16,18,20–24^. The MB-based lung cancer panel targeted 17 genes containing 62 primer pairs and spanned 6.4 kb (Table 1). The entire exons of *TP53* were covered by both the MB-based biliary-pancreatic and lung panels.

**Table 1:** Gene lists for molecular barcode and non-molecular barcode sequencing.

**Non-MB-based biliary-pancreatic panel (60 genes)**
*ACVR1B, ACVR2A, AKT1, APC, ARID1A, ARID1B, ARID2, ATM, AXIN1, BAP1, BRAF, BRCA1, BRCA2, CDKN2A, CTNNB1, EGFR, ELF3, EPC1, ERBB2, ERBB3, ERBB4, FGFR2, GNAS, HRAS, IDH1, IDH2, JAK3, KMT2C(MLL3), KRAS, MAP2K4, MAP2K7, MAPK10, MLH1, MLL, MSH2, MSH6, MYC, NF1, NFE2L2, NRAS, NRG1, PALB2, PBRM1, PIK3CA, PMS2, PTEN, RBM10, RNF43, ROBO1, ROBO2, SF3B1, SLIT2, SMAD4, SOS2, SRC, STK11, TGFBR2, TP53, TSC1, TSC2*
**MB-based biliary-pancreatic panel (21 genes)**
*AKT1, APC, AXIN1, BRAF, CTNNB1, EGFR, ELF3, ERBB2, FBXW7, GNAS, HRAS, IDH1, IDH2, KRAS, MAP2K1, NF1, NFE2L2, NRAS, PIK3CA, SMAD4, TP53*
**Non-MB-based lung cancer panel (53 genes)**
*AKT1, AKT2, AKT3, ARID1A, ARID1B, ARID2, ASCL4, ATM, BRAF, CDKN2A, COBL, CREBBP, CTNNB1, CUL3, EGFR, EP300, EPHA7, ERBB2, ERBB3, FGFR1, FGFR2, FGFR3, FOXP2, HRAS, KEAP1, KMT2A, KMT2D, KRAS, MAP2K1, MET, MGA, NF1, NFE2L2, NOTCH1, NOTCH2, NRAS, PIK3CA, PTEN, RASA1, RB1, RBM10, RIT1, SETD2, SLIT2, SMAD4, SMARCA4, SOX2, STK11, TP53, TP63, TSC1, TSC2, U2AF1*
**MB-based lung cancer panel (17 genes)**
*AKT1, ALK, BRAF, CTNNB1, EGFR, ERBB2, HRAS, KRAS, MAP2K1, MET, MET, NFE2L2, NRAS, PIK3CA, ROS1, TP53, U2AF1*

We searched the literature and selected genes based on the following criteria: (a) significantly mutated genes relative to the background mutation rates analyzed by MutSigCV analysis tool; (b) genes involved in signaling pathways and potential therapeutic targets in biliary-pancreatic or lung cancers; and (c) known driver genes or tumor suppressor genes reported by TCGA^25,26^ and another research institute^27–32^. We examined the hotspot mutation site of each gene from the Catalogue Of Somatic Mutations In Cancer (COSMIC) database^33^. Based on these previous data, we analyzed frequently mutated genes and known somatic variants using tumor-normal pair samples.

### Non-MB based library preparation

Targeted sequencing was performed as previously described^23,34–37^. Multiplex PCR was performed using the Ion AmpliSeq Library Kit Plus and Ion AmpliSeq primer (Thermo Fisher Scientific) at 99 °C for 2 min, followed by 14–18 cycles of 99 °C for 15 s and 60 °C for 4 min, with a final hold at 10 °C. Primer sequences were partially digested with FuPa reagent at 50 °C for 10 min, followed by 55 °C for 10 min and 60 °C for 20 min. Adaptor and barcode ligation was performed using Ion Xpress Barcode Adapters at 22 °C for 30–60 min, 68 °C for 5 min, 72 °C for 5 min and hold at 10 °C.

### MB-based library preparation

Multiplex PCR was performed with Ion AmpliSeq HD primer and Ion AmpliSeq HD Library Kit (Thermo Fisher Scientific) in accordance with the manufacturer’s instruction. Primer sets comprised two different primer pools. The reaction mixture comprised 3.7 μL of 4x Amplification Mix, 1.5 μL of 10x forward primer mix, 1.5 μL of 10x reverse primer mix, 1–20 ng of FFPE or plasma DNA, and nuclease-free water up to 15 μL of total volume. PCR was performed to amplify the target regions with the following cycling conditions: three cycles of 99 °C for 30 s, 64 °C for 2 min, 60 °C for 6 min and 72 °C for 30 sec, 72 °C for 2 min with a final hold at 4 °C. After combining the PCR products, amplicons were partially digested with 5 μL of SUPA reagent. Reactions were performed using the following conditions: 30 °C for 15 min, 50 °C for 15 min, 55 °C for 15 min, 25 °C for 10 min, 98 °C for 2 min and hold at 4 °C. Libraries were amplified with 4 μL of Ion AmpliSeq HD Dual Barcode Kit with the following condition: 99 °C for 15 s, 5 cycles of 99 °C for 15 s, 62 °C for 20 s and 72 °C for 20 s, 15–17 cycles of 99 °C for 15 sec and 70 °C for 40 sec, and 72 °C for 5 min and hold at 4 °C.

### Single amplicon targeted sequencing of individual discordant mutations sites

There were two mutations observed in tumor samples by Non-MB sequencing, but not by MB sequencing (*SMAD4* p. R97H in case #1 and *TP53* p.G244S in case #10). For confirmation of these discordant results, we amplified the mutations sites with specific primers as follow: 5′-GTGGCTGGTCGGAAAGGATT-3′ and 5′-CCAGGTGATACAACTCGTTCG-3′ for *SMAD4* p.R67H; 5′-TGATGATGGTGAGGATGGGC-3′ and 5′-CTGCTTGCCACAGGTCTCC-3′ for *TP53* p.G244S. PCR was performed with PrimeSTAR HS DNA Polymerase (TaKaRa Bio, Shiga, Japan). PCR products were visualized by agarose gel electrophoresis and purified with Agencourt AMPure XP reagents (Beckman Coulter, Brea, CA, USA). End repair and barcode adaptors were ligated with Ion Plus Fragment Library Kit in accordance with the manufacturer’s instructions to construct libraries.

### Library purification and sequencing

Library purification was performed using Agencourt AMPure XP reagents (Beckman Coulter) on KingFisher Duo Prime. The library concentration was determined using an Ion Library TaqMan Quantitation Kit (Thermo Fisher Scientific); each library was diluted to 50–60 pM, and the same amount of libraries was pooled for one sequence reaction. Emulsion PCR and chip loading was performed on the Ion Chef with the Ion 540 Kit-Chef or Ion PI Hi-Q Chef kit; sequencing was performed using Ion 540 Kit-Chef on the Ion GeneStudio S5 Prime System or Ion PI Hi-Q Sequencing Kit on an Ion Proton Sequencer (Thermo Fisher Scientific).

### Data analysis

Sequence data were processed using standard pipeline in Torrent Suite Software running on the Torrent Server. Raw signal data were analyzed using Torrent Suite version 5.10.0. The data processing pipeline involved signaling processing, base calling, quality score assignment, adapter trimming, PCR duplicate removal, read alignment to the human genome 19 reference (hg19), quality control of mapping quality, coverage analysis, and variant calling. Following data analysis, the annotation of single nucleotide variants, insertions, and deletions was performed by the Ion Reporter Server System (Thermo Fisher Scientific). Binary SAM (BAM) files were visualized by Ion Reporter Genomic Viewer to check the variant in plasma.

For Ion AmpliSeq panel analysis (Non-MB method), buffy coat DNA was used as a control to detect confident variants in tumors (Tumor–Normal pairs). We used the following filtering parameters for variant calling: (i) the minimum number of variant allele reads was ≥10, (ii) the coverage depth was ≥20, (iii) UCSC Common single nucleotide polymorphisms (SNPs) = Not In, and (iv) Confident Somatic Variants = In.

For Ion AmpliSeq HD panel analysis (MB method), variants were detected using the workflow of “AmpliSeq HD for Liquid Biopsy w2.1 - DNA - Single Sample” with minor modification. We changed parameters in variant finding as follows: (i) minimum number of SNP variant supporting functional families = 2, (ii) minimum number of hotspot variant supporting functional families = 2, (iii) minimum number of reads with same unique molecular tag (UMT) required to form a functional family = 2, (iv) minimum number of insertion and deletion (INDEL) variant supporting functional families = 6, and (v) require family of size to be functional for calling homopolymer INDEL = 2. We used the following filtering parameters for variant calling: (i) Alternate Allele Count ≥2, (ii) UCSC Common SNPs = Not In, (iii) p-value<0.02, (iv) exclude INDEL variants with less than VAF < 0.002 or the number of mutated alleles ≤ 3, and (v) exclude non-hotspot variants with the number of mutated alleles ≤3. To detect variants in tumors by MB sequencing, the VAF cut-off was ≥0.005. Identical mutations corresponding to tumor DNA were called in plasma DNA. Sequence data were visually confirmed with the Ion Reporter Genomic Viewer and any sequence, alignment, or variant call error artifacts were discarded. Pathogenic variants were annotated with the OncoKB database^38^.

### Refine the mapping condition

If there was a discordance in the VAFs between the MB and non-MB data in the tumour, manual review of the mapping conditions was performed. We observed two alterations near in *TP53* gene (c.986 C > G, p.T329S; c.956_978delAGAAGAAACCACTGGATGGAGAA, p.K319fs) in case #8. We visualized BAM files by IGV and refined the mapping conditions (Supplemental Fig. 1A–C). Both two variants exists in the same sequencing reads. The data possibly showed that FuPa treatment removed the primer part from the most sequence reads generated by Non-MB, whereas primer part were remains in the reads generated by MB. When Non-MB data was mapped with default conditions, remaining primer parts would affect the mapping status (Supplemental Fig. 1A). Actually, 3′-end of reverse strand reads from the deletion site were not mapped based on alignment scoring condition. The sequencing reads without primer part contained “soft-clip” sequence and were not aligned (Supplemental Fig. 1A). On the other hand, reads with the remaining primer part had a longer 3′-end strand and the entire sequence reads with the deletion were aligned. When we modified the mapping parameters to allow long insertion/deletion, the VAF increased from 26% to 42.6% (Supplemental Fig. 1B). Before classification of molecular families based on MB, the raw data of sequence read showed VAF was 49% (Supplemental Fig. 1C).

### Ethical approval

All procedures performed in studies involving human participants were in accordance with the ethical standards of the institutional clinical research and genome research committee at Yamanashi Central Hospital (G-2018-1) and with the 1964 Helsinki declaration and its later amendments or comparable ethical standards.

### Informed consent

Informed consent was obtained from all individual participants included in the study.

## Results

### Amplicon-based target enrichment with MB

**Figure 1:**
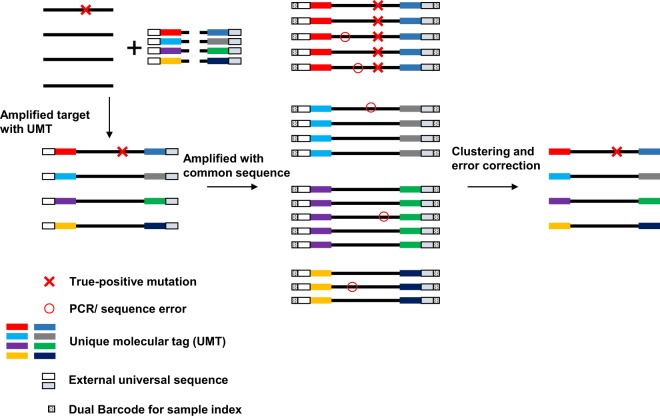
Workflow of library construction and molecular barcode sequencing. Primers harboring the unique molecular tag (UMT) were used to amplify the target regions of interest. UMT is a DNA sequence and serves as a molecular barcode that distinguish each template DNA. UMT is tagged the 5′ and 3′ end of template DNA. After the first amplification, second amplification is performed using an external universal sequence. Molecular barcodes are used to cluster multiple reads that originated from the same template DNA for error correction. Sequence/PCR errors are eliminated for subsequent mutational calling analysis.

FFPE DNA used for PCR amplification was treated with uracil DNA glycosylase to remove deaminated cytosines and reduce the artificial error (C to T conversion) in variant calling. Template DNA was subjected to the first PCR amplification using Ion AmpliSeq HD primers containing a UMT, followed by second PCR amplification using an external universal sequence (Fig. 1). After amplicons were partially digested, libraries were amplified and dual-barcoded at both the 5′ and 3′ ends. Dual-barcode was used to increase the specificity using end-to-end analyzed sequence reads for subsequent analysis. The identical UMT-ligated reads were clustered into molecular family. A true-positive somatic mutation was called when the mutation was presented in all reads among molecular families (Fig. 1).

To validate the analytical performance, we conducted serial dilution assay using standard cfDNA containing *EGFR* variants purchased from Horizon. Our designed MB-based lung cancer panel covers eight *EGFR* variants. Except for *EGFR* V769-D770insASV, we identified seven variants down to 0.5% (Table 2). We also found five mutations which harbored 0.25% expected VAF.

**Table 2:** Serial dilution analysis with cfDNA.

Gene	Variant	Expected VAF					
		0.1%	0.25%	0.5%	1%	2.5%	5%
*EGFR*	L858R	ND	1.38	0.61	1.17	1.72	7.96
*EGFR*	ΔE746-A750	ND	0.54	1.41	0.61	2.39	5.52
*EGFR*	T790M	ND	0.43	0.79	0.87	3.33	5.93
*EGFR*	V769-D770insASV	ND	ND	ND	ND	ND	0.25
*EGFR*	L861Q	ND	1.72	0.61	1.56	0.92	6.47
*EGFR*	G719S	ND	0.98	0.8	1.94	1.31	5.04
*EGFR*	C797S	ND	ND	0.34	0.43	0.64	2.06
*EGFR*	S768I	0.32	ND	0.62	2.12	2.11	6.03

ND, not detected.

### Mutations in plasma cfDNA detected by non-MB and MB sequencing

We performed targeted sequencing with Non-MB and MB technologies using a total of 24 samples (12 tumors and 12 plasma) from 12 patients. The median coverage depths were 911× (range: 184–4,634×) and 43,117× (range: 6,762–128,044×) by Non-MB and MB sequencing, respectively (Supplemental Table 2). The median on target rates (sequencing read mapped on target region) were 91.0% (62.0–97.7%) and 97.9% (96.1–99.1%) and the uniformities were 88.1% (64.6–96.6%) and 96.9% (92.1–100.0%), respectively (Supplemental Table 2).

**Figure 2:**
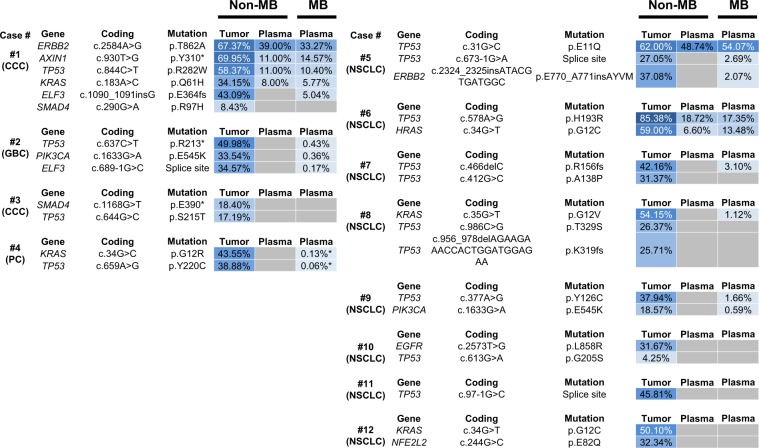
Molecular barcode sequencing sensitively detects the tumor-derived mutations in plasma cell free DNA (cfDNA). Heat map shows the mutation profiles in tumors and plasma cfDNA using both sequencing methods. Samples were collected from 12 patients with biliary-pancreatic (Case #1–4) and non-small cell lung cancers (Case #5–12). Sequencing was performed with non-molecular barcode (Non-MB) and molecular barcode (MB) technologies. Identical mutations in plasma cfDNA corresponding to mutations in tumor samples were detected. Variant allele fraction (%) is shown in each box and is indicated by the graduated color scale from 1% (light blue) to 100% (dark blue). Gray box indicates no identified alterations. Tumor types (CCC, cholangiocarcinoma; GBC, gallbladder cancer; PC, pancreatic cancer, NSCLC, non-small cell lung cancer) were denoted under the case number. Variant with asterisk (*) shows the mutations which are detected by visual inspection of Binary SAM (BAM) files by Ion Reporter Genomic Viewer.

**Figure 3:**
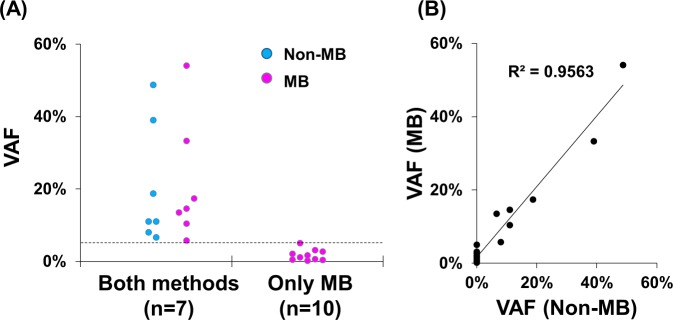
Molecular barcode sequencing detects low levels of mutated allele in plasma cfDNA. (**A**) Graph of variant allele fraction (VAF) of each mutation according to sequencing method. Both molecular barcode (MB) and non-molecular barcode (Non-MB) detected 7 mutations in plasma cfDNA. Ten mutations were detected by only MB sequencing. Dot line shows 5% VAF which is the detection limit of Non-MB seq. (**B**) Dot plot of the VAF in plasma cfDNA between MB and Non-MB sequencing. Mutations were detected with a high level of accuracy and concordance (decision coefficient, R^2^ = 0.9563).

First, we examined genetic alterations in tumors from patients with biliary-pancreatic cancer (Case #1–4) and non-small cell lung cancer (Case #5–12) by Non-MB sequencing (Fig. 2). We identified a total of 30 somatic mutations in tumors by the Non-MB method. We next investigated whether identical mutations corresponding to tumors were observed in plasma cfDNA. Non-MB and MB sequencing identified 7 and 17 mutations in plasma, respectively (Fig. 2). At least one mutation was identified in plasma of 3 (25%) and 7 (58%) patients by Non-MB and MB sequencing by the Ion Reporter pipeline, respectively. Seven mutations in plasma cfDNA were identified by both methods and VAFs of these mutations were above 5%. Notably, MB sequencing could detect tumor-derived mutations with less than about 5% in plasma cfDNA. Thus, MB technology enabled the detection of mutations harboring low VAF down to 0.17% in plasma cfDNA (Fig. 3A). By visual inspection of Binary SAM (BAM) files by Ion Reporter Genomic Viewer, we observed additional two mutations in plasma cfDNA in case #4. The VAF of these two mutations were 0.13% (4 out of 3,053 reads) in *KRAS* p.G12R and 0.06% (6 out of 9,859 reads) in *TP53* p.Y220C (Fig. 2). These results suggested that MB sequencing could sensitively detect the somatic mutations derived from tumors in plasma cfDNA. Additionally, the observed VAFs in plasma cfDNA were correlated with high accuracy between Non-MB and MB sequencing (R^2^ = 0.9563) (Fig. 3B).

### Clonal and subclonal mutations analyzed by MB sequencing

**Figure 4:**
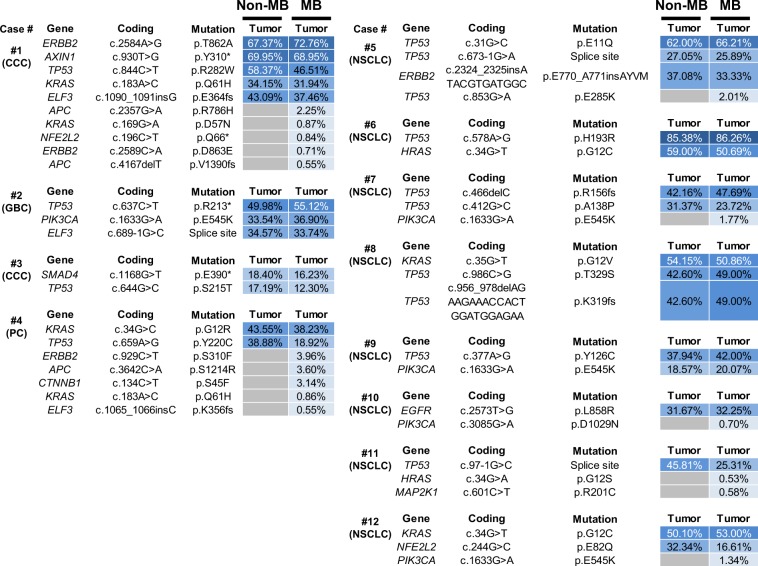
Molecular barcode sequencing revealed mutations in clonal and subclonal tumor population. Heat map shows the mutation profiles in tumors according to sequencing method. Samples were collected from the same patients described in Fig. 2. MB sequencing identified clonal and subclonal mutations in tumors. MB and Non-MB sequencing identified the same mutations with high variant allele fraction (VAF). VAF (%) is indicated in each box. Variant allele fractions are indicated in the graduated color scale from 1% (light blue) to 100% (dark blue). Gray box indicates no identified alterations. Tumor types (CCC, cholangiocarcinoma; GBC, gallbladder cancer; PC, pancreatic cancer, NSCLC, non-small cell lung cancer) were denoted under the case number.

We identified a total of 30 and 44 somatic mutations in tumors by Non-MB and MB sequencing, respectively (Fig. 4). Two variants were identified by Non-MB sequencing in Case 1 (*SMAD4*, p.R97H, c.290 G > A, VAF = 8.43%) and Case 10 (*TP53*, p.G244S, c.730 G > A, VAF = 4.25%). To examine whether these variants were false-positive results, we performed amplicon ultra-deep sequence using PCR products. We obtained high-depth mapped data and visually confirmed these mutations. The data showed the VAF of *SMAD4* p.R97H mutation was 0.0036% (variant reads: total reads, 9: 249,780) (Supplemental Fig. 2) and that of *TP53* p.G244S was 0.91% (2,145: 236,446) (Supplemental Fig. 3). Because these VAF value was not consistent with the result of Non-MB sequencing, we considered these two variants were artefacts. Although the number *TP53* mutant variant was relatively high (2,145 reads), maybe due to the artefact (*e.g*. C > T conversion) during formalin fixation. These two variants were excluded from the subsequent results.

Both methods detected the same 28 mutations with over 10% VAF (VAF = 12.30–86.26%). The 28 mutations were in *TP53* (n = 13), *KRAS* (n = 4), *PIK3CA* (n = 2), *ERBB2* (n = 2), *ELF3* (n = 2), *SMAD4* (n = 1), *AXIN1* (n = 1), *EGFR* (n = 1), *NFE2L2* (n = 1) and *HRAS* (n = 1). Of these 28 mutations, 25 (89%) were annotated as pathogenic mutations by OncoKB database^38^, indicating these were clonal driver mutations.

**Figure 5:**
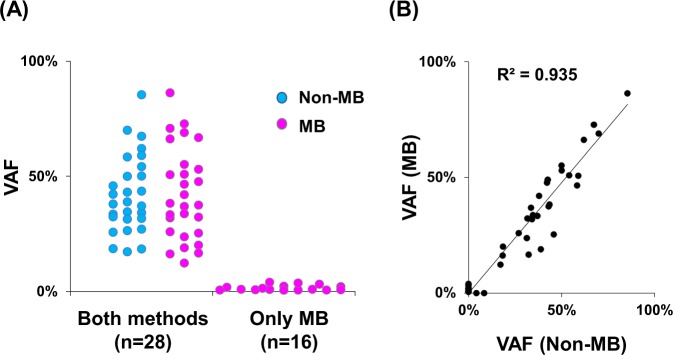
Molecular barcode sequencing detects low mutated allele in tumor DNA. (**A**) Plot of the variant allele fraction (VAF) of each mutation according to sequencing method. Twenty-eight mutations were detected by both molecular barcode (MB) and non-molecular barcode (Non-MB) sequencing, whereas 16 mutations were identified by only MB sequencing. **(B)** Dot plot of VAF in tumors between MB and Non-MB sequencing. Mutations were detected with correlation after refining the mapping condition (decision coefficient, R^2^ = 0.935).

Tumors acquire somatic mutations during tumor evolution and are comprised of clonal and subclonal clones. Because MB sequencing could detect low VAF mutations, we reasoned that subclonal mutations were detected by MB technology. Sixteen mutations (VAF: 0.53–3.96%) were detected only by MB (Figs. 4 and 5A). Among these 16 mutations, 10 (63%) were putative pathogenic mutations. Subclonal pathogenic mutations were identified, such as *ERBB2* p.S310F (VAF = 3.96%), *CTNNB1* p.S45F (3.14%), *KRAS* p.Q61H (0.86%), *ELF3* frameshift (0.55%) and *APC* frameshift (0.55%) in biliary-pancreatic tumors and *TP53* p.E285 (2.01%), *PIK3CA* p.E545K (1.77% and 1.34%), *PIK3CA* p.D1029N (0.70%), and *HRAS* p.G12C (0.53%) in non-small cell lung cancers. Notably, *PIK3CA* mutations were frequently annotated as subclonal driver mutations in 38% (3/8) of patients with non-small cell lung cancer^39^. These results suggested that the subclonal driver mutations reflected the tumor heterogeneity. The observed VAF between Non-MB and MB sequencing were correlated with high accuracy (R^2^ = 0.935) (Fig. 5B).

## Discussion

In this study, we evaluated the performance of Non-MB and MB sequencing for detecting somatic mutations in tumors and plasma cfDNA by comparing the MB sequencing data using IonAmpliSeq HD with the results obtained by Non-MB sequencing. The MB sequencing identifies rare variants and shows benefits for analyzing tumor heterogeneity. These results suggested that MB sequencing can be applied for detecting low levels of mutated alleles in the presence of high amounts of wild-type allele.

Several intrinsic or acquired rare variants in cancer have been identified that are associated with drug resistance. For instance, *EGFR* T790M and C797S and *ALK* L1196M mutations are associated with resistance to EGFR tyrosine kinase inhibitor and ALK inhibitors in non-small cell lung cancer, respectively^40–43^. Subpopulational *KRAS* activating mutations are associated with resistance to anti-EGFR therapy (e.g. cetuximab and panitumumab) in colorectal cancer^44,45^. Furthermore, activating mutations in *ERBB2*, which encodes HER2, and *ESR* mutations in the ligand binding domain were identified in non-responding breast cancer patients treated with anti-HER2 antibodies and hormone therapy^46–48^. Some of these resistant variants emerged after drug treatment^45^. Previous studies suggested that monitoring resistance mutations in plasma cfDNA is useful for the evaluation of treatment effects^16,45,49^. Our analysis showed that tumor-derived mutations with low VAF (approximately 0.17%) were successfully detected in plasma cfDNA by MB-based sequencing. These mutations with low VAF would not be detectable by Non-MB sequencing, because the false-positive rate might be increased when the threshold for variant finding was lowered. Therefore, MB sequencing with high-depth coverage would eliminate error-prone nucleotide changes. MB sequencing enable researchers to identify resistance-related variants at low levels.

Tumors comprise clonal and subclonal populations that are acquired during tumor evolution^1^. Driver genes mainly occur at the early phase of tumorigenesis and occupy a major cell population within tumors. Interestingly, a previous study showed that well-known driver mutations may occur at late phase of tumor progression as subclonal mutations^2^. The Tracking Non-Small-Cell Lung Cancer Evolution through Therapy (TRACERx) study revealed that more than 75% of the tumors carried a subclonal driver alteration^39^. Some of these subclonal driver mutations were therapeutic targets. Whether molecular-targeting drugs are effective to tumors containing subclonal population harboring “actionable” mutations is an important question. As previous report^43,50,51^, our data showed that MB sequencing also detected *PIK3CA, ERBB2* and *KRAS* subclonal mutations, which are linked to therapy response in various cancers. Thus, MB sequencing with Ion Ampliseq HD technology will be useful to detect subclonal mutations related to cancer therapy response.

The preparation of a MB-ligated library using IonAmpliSeq HD was a simple procedure (Fig. 1). This process required three steps and took approximately 3 h: a first amplification of target regions with custom designed MB primers (~1 h), partial digestion of primers (~1 h) and a second amplification with barcode for sample index (~1 h). The hands-on time was approximately 45–60 min. However, MB-based sequencing has some limitations. The target region coverage should be narrowed down to achieve high-depth coverage. Output sequencing data from Ion Proton and Ion GeneStudio S5 systems is 10–15 G bp. To analyze multiple samples and reduce the running cost, target regions should be selective. However, a high-throughput sequencing machine that can yield more reads will resolve this problem.

In conclusion, here we demonstrated that the MB sequencing technology was useful to detect rare variants in tumor and liquid biopsy. This technology will be a powerful tool for analyzing tumor heterogeneity, somatic mosaicism, and tumor-derived DNA in liquid biopsies (*e.g*. plasma). Furthermore, Ion Ampliseq HD technology will be an alternative MB sequencing method and enable the investigation of expectedly low VAF mutations in tumors and plasma.

## Supplementary information

Supplementary information.

## References

[1] McGranahan N, Swanton C (2017). Clonal Heterogeneity and Tumor Evolution: Past, Present, and the Future. Cell.

[2] McGranahan N (2015). Clonal status of actionable driver events and the timing of mutational processes in cancer evolution. Sci. Transl. Med..

[3] Bettegowda C (2014). Detection of circulating tumor DNA in early- and late-stage human malignancies. Sci. Transl. Med..

[4] Salk JJ, Schmitt MW, Loeb LA (2018). Enhancing the accuracy of next-generation sequencing for detecting rare and subclonal mutations. Nat. Rev. Genet..

[5] Luthra R (2017). A Targeted High-Throughput Next-Generation Sequencing Panel for Clinical Screening of Mutations, Gene Amplifications, and Fusions in Solid Tumors. J. Mol. Diagn..

[6] Mehrotra M (2017). Study of Preanalytic and Analytic Variables for Clinical Next-Generation Sequencing of Circulating Cell-Free Nucleic Acid. J. Mol. Diagn..

[7] Demuth C, Winther-Larsen A, Madsen A, Meldgaard P, Sorensen B (2018). A Method for Treatment Monitoring Using Circulating Tumour DNA in Cancer Patients Without Targetable Mutations. Oncotarget.

[8] Kinde I, Wu J, Papadopoulos N, Kinzler K, Vogelstein B (2011). Detection and quantification of rare mutations with massively parallel sequencing. Proc. Natl Acad. Sci. USA.

[9] Newman AM (2016). Integrated digital error suppression for improved detection of circulating tumor DNA. Nat. Biotechnol..

[10] Schmitt M (2012). Detection of ultra-rare mutations by next-generation sequencing. Proc. Natl Acad. Sci. USA.

[11] Corcoran RB, Chabner BA (2018). Application of Cell-free DNA Analysis to Cancer Treatment. N. Engl. J. Med..

[12] Wan JCM (2017). Liquid biopsies come of age: towards implementation of circulating tumour DNA. Nat. Rev. Cancer.

[13] Amemiya K (2016). Touch imprint cytology with massively parallel sequencing (TIC-seq): a simple and rapid method to snapshot genetic alterations in tumors. Cancer Med..

[14] Sakamoto I (2016). BRCA1 and BRCA2 mutations in Japanese patients with ovarian, fallopian tube, and primary peritoneal cancer. Cancer.

[15] Hirotsu Y (2015). Detection of BRCA1 and BRCA2 germline mutations in Japanese population using next-generation sequencing. Mol. Genet. Genomic Med..

[16] Iijima Y (2017). Very early response of circulating tumour-derived DNA in plasma predicts efficacy of nivolumab treatment in patients with non-small cell lung cancer. Eur. J. Cancer.

[17] Amemiya K, Hirotsu Y, Oyama T, Omata M (2019). Relationship between formalin reagent and success rate of targeted sequencing analysis using formalin fixed paraffin embedded tissues. Clin. Chim. Acta.

[18] Goto T, Hirotsu Y, Oyama T, Amemiya K, Omata M (2016). Analysis of tumor-derived DNA in plasma and bone marrow fluid in lung cancer patients. Med. Oncol..

[19] Jiang P (2015). Lengthening and shortening of plasma DNA in hepatocellular carcinoma patients. Proc. Natl Acad. Sci. USA.

[20] Goto T (2017). Detection of tumor-derived DNA dispersed in the airway improves the diagnostic accuracy of bronchoscopy for lung cancer. Oncotarget.

[21] Goto T (2017). Distribution of circulating tumor DNA in lung cancer: analysis of the primary lung and bone marrow along with the pulmonary venous and peripheral blood. Oncotarget.

[22] Goto T (2017). Stepwise addition of genetic changes correlated with histological change from “well-differentiated” to “sarcomatoid” phenotypes: a case report. BMC Cancer.

[23] Goto T (2017). Mutational analysis of multiple lung cancers: Discrimination between primary and metastatic lung cancers by genomic profile. Oncotarget.

[24] Nakagomi T (2018). New therapeutic targets for pulmonary sarcomatoid carcinomas based on their genomic and phylogenetic profiles. Oncotarget.

[25] Cancer Genome Atlas Research Network (2017). Integrated Genomic Characterization of Pancreatic Ductal Adenocarcinoma. Cancer Cell.

[26] Farshidfar F (2017). Integrative Genomic Analysis of Cholangiocarcinoma Identifies Distinct IDH-Mutant Molecular Profiles. Cell Rep..

[27] Jiao Y (2013). Exome sequencing identifies frequent inactivating mutations in BAP1, ARID1A and PBRM1 in intrahepatic cholangiocarcinomas. Nat. Genet..

[28] Nakamura H (2015). Genomic spectra of biliary tract cancer. Nat. Genet..

[29] Bailey P (2016). Genomic analyses identify molecular subtypes of pancreatic cancer. Nat..

[30] Scarpa A (2017). Whole-genome landscape of pancreatic neuroendocrine tumours. Nat..

[31] Jiao Y (2014). Whole-exome sequencing of pancreatic neoplasms with acinar differentiation. J. Pathol..

[32] Yachida S (2016). Genomic Sequencing Identifies ELF3 as a Driver of Ampullary Carcinoma. Cancer Cell.

[33] Tate JG (2019). COSMIC: the Catalogue Of Somatic Mutations In Cancer. Nucleic Acids Res..

[34] Hirotsu Y (2016). Comparison between two amplicon-based sequencing panels of different scales in the detection of somatic mutations associated with gastric cancer. BMC Genomics.

[35] Hirotsu Y (2015). Multigene panel analysis identified germline mutations of DNA repair genes in breast and ovarian cancer. Mol. Genet. Genomic Med..

[36] Takaoka S (2019). Molecular subtype switching in early-stage gastric cancers with multiple occurrences. J. Gastroenterol..

[37] Hirotsu Y (2016). Targeted and exome sequencing identified somatic mutations in hepatocellular carcinoma. Hepatol. Res..

[38] Chakravarty, D. *et al*. OncoKB: A Precision Oncology Knowledge Base. *JCO Precision Oncology* (2017). 10.1200/PO.17.00011 PMC5586540 28890946

[39] Jamal-Hanjani M (2017). Tracking the Evolution of Non-Small-Cell Lung Cancer. N. Engl. J. Med..

[40] Zhou W (2009). Novel mutant-selective EGFR kinase inhibitors against EGFR T790M. Nat..

[41] Thress KS (2015). Acquired EGFR C797S mutation mediates resistance to AZD9291 in non-small cell lung cancer harboring EGFR T790M. Nat. Med..

[42] Choi Y (2010). EML4-ALK mutations in lung cancer that confer resistance to ALK inhibitors. N. Engl. J. Med..

[43] Piotrowska, Z. *et al*. Heterogeneity and Coexistence of T790M and T790 Wild-Type Resistant Subclones Drive Mixed Response to Third-Generation Epidermal Growth Factor Receptor Inhibitors in Lung Cancer. *JCO Precis Oncol* (2018). 10.1200/PO.17.00263 PMC6097183 30123863

[44] Morelli MP (2015). Characterizing the patterns of clonal selection in circulating tumor DNA from patients with colorectal cancer refractory to anti-EGFR treatment. Ann. Oncol..

[45] Misale S (2012). Emergence of KRAS mutations and acquired resistance to anti-EGFR therapy in colorectal cancer. Nat..

[46] Bose R (2013). Activating HER2 mutations in HER2 gene amplification negative breast cancer. Cancer Discov..

[47] Hirotsu Y (2017). Intrinsic HER2 V777L mutation mediates resistance to trastuzumab in a breast cancer patient. Med. Oncol..

[48] Robinson DR (2013). Activating ESR1 mutations in hormone-resistant metastatic breast cancer. Nat. Genet..

[49] Schiavon G (2015). Analysis of ESR1 mutation in circulating tumor DNA demonstrates evolution during therapy for metastatic breast cancer. Sci. Transl. Med..

[50] Turke AB (2010). Preexistence and clonal selection of MET amplification in EGFR mutant NSCLC. Cancer Cell.

[51] Van Emburgh BO (2016). Acquired RAS or EGFR mutations and duration of response to EGFR blockade in colorectal cancer. Nat. Commun..

