# Supplementary material for: Dual-molecular barcode sequencing detects rare variants in tumor and cell free DNA in plasma
Source: Sci Rep. 2020 Feb 25;10:3391. doi: 10.1038/s41598-020-60361-3 (PMC7042261; doi:10.1038/s41598-020-60361-3)
Supplement: Supplementary file 1 — Supplementary information. [file 41598_2020_60361_MOESM1_ESM.pdf]

Dual-molecular barcode sequencing detects rare variants in tumor and cell free DNA in plasma

Yosuke Hirotsu[1], Sotaro Otake[2], Hiroshi Ohyama[3,4], Kenji Amemiya[1], Rumi Higuchi[2], Toshio Oyama[5], Hitoshi Mochizuki[1,3], Taichiro Goto[2] and Masao Omata[3,6]

[1]Genome Analysis Center, [2]Lung Cancer and Respiratory Disease Center, [3]Department of Gastroenterology, Yamanashi Central Hospital, 1-1-1 Fujimi, Kofu, Yamanashi, Japan
[4]Department of Gastroenterology, Chiba University, Graduate School of Medicine, Inohana, Chuo-ku, Chiba, Japan
[5]Department of Pathology, Yamanashi Central Hospital, 1-1-1 Fujimi, Kofu, Yamanashi, Japan
[6]The University of Tokyo, 7-3-1 Hongo, Bunkyo-ku, Tokyo, Japan

*Correspondence to: Yosuke Hirotsu, Genome Analysis Center, Yamanashi Central Hospital, Kofu, Japan
Email: hirotsu-bdyu@ych.pref.yamanashi.jp
Tel: +81-55-253-7111, Fax: +81-55-253-8011

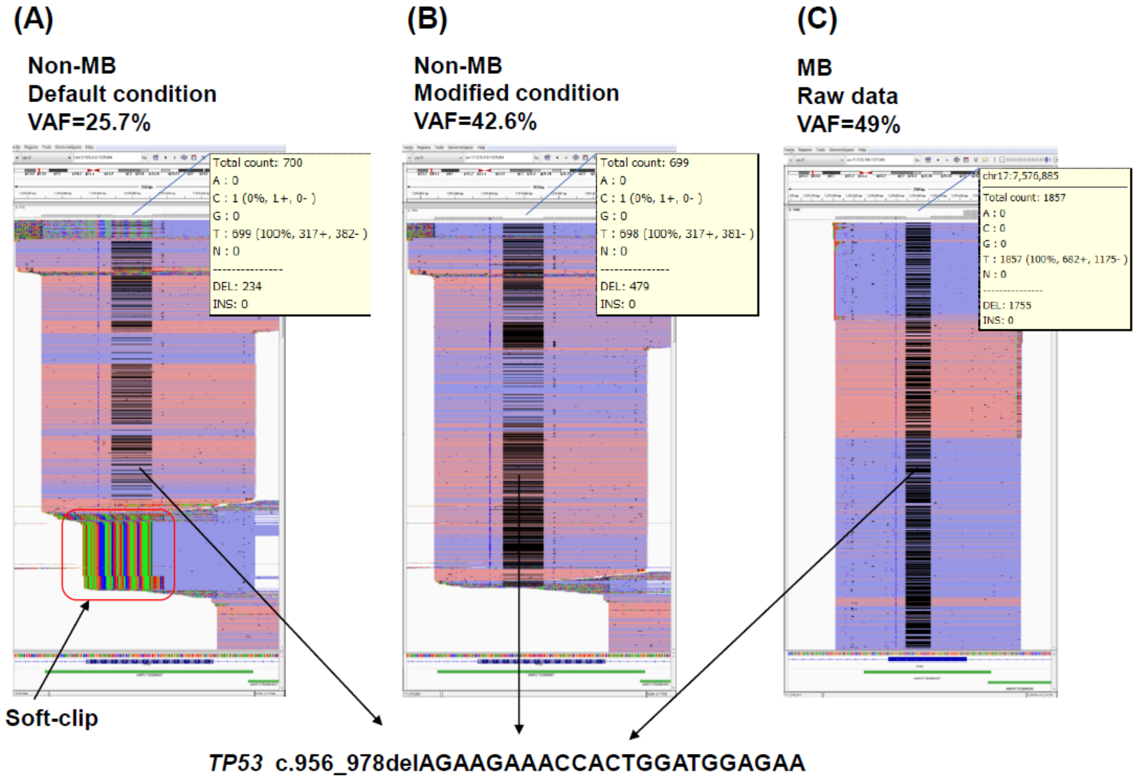

Supplemental Figure 1

Modified mapping condition improves the sequencing data. **(A-C)** BAM files of non-molecular barcode (Non-MB) and molecular barcode (MB) sequencing were visualized by IGV. Individual forward and reverse sequencing reads were represented by red and purple horizontal lines, respectively. Black line represents the 23 bp deletion mutation at *TP53* (c.956_978delAGAAGAAACCACTGGATGGAGAA) **(A)** Sequence reads are aligned with default condition. Soft clipped bases appear as multi-colour in the red line. **(B)** After applied modified condition, full length reads are mapped on the reference. **(C)** Raw data of MB sequence was visualized. VAF, variant allele fraction.

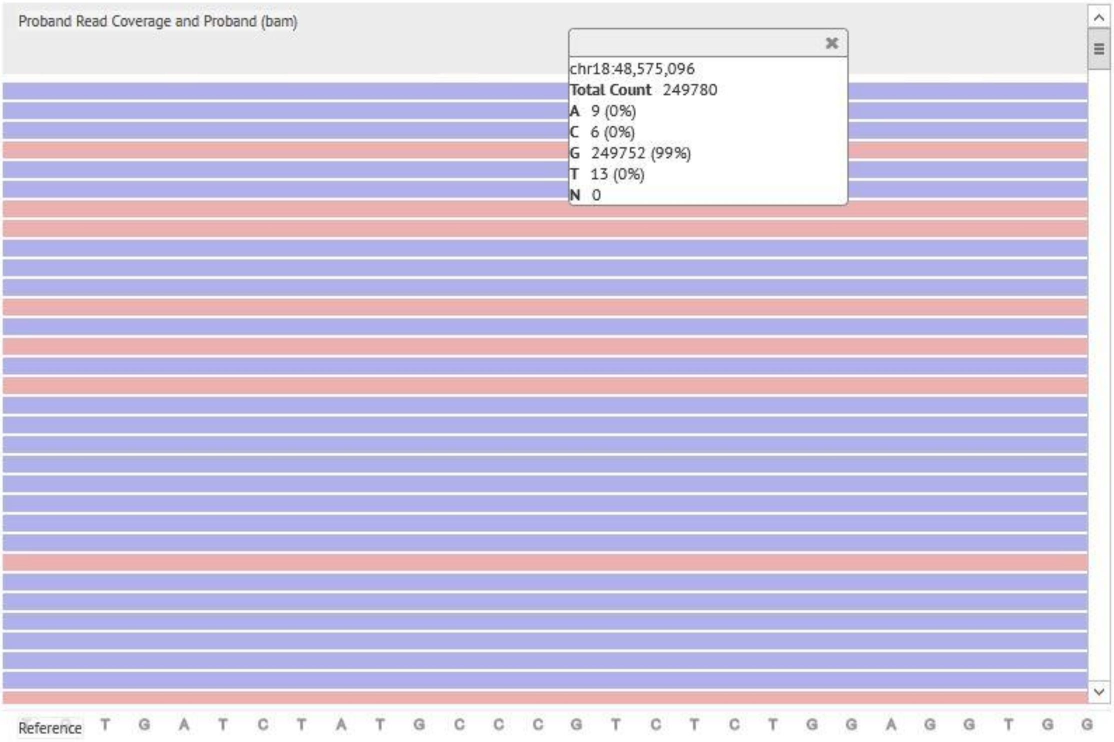

Supplemental Figure 2

Amplicon ultra-deep sequencing data is visualized by Ion Reporter Genome Viewer.

The variant allele fraction is 0.0036% at the location (chr18:48,575,096: *SMAD4,* c.290G>A, p.R97H).

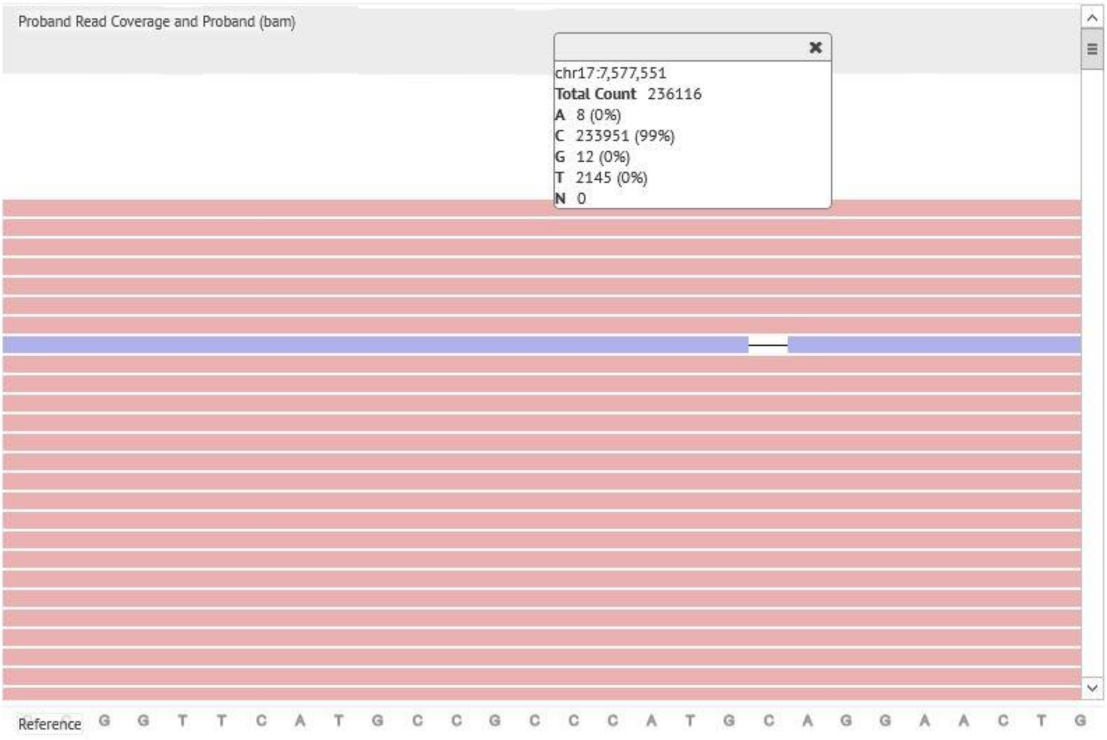

Supplemental Figure 3

Amplicon ultra-deep sequencing data is visualized by Ion Reporter Genome Viewer.

The variant allele fraction is 0.91% at the location (chr17:7,577,551: *TP53,* c.730G>A p.G244S).

Supplemental Table 1. Patient characteristics and sample information

Case number	Tumor type	Tumor size (mm)	Stage	Sample description	The fraction of tumour cells	Timing of plasma sampling
#1	Cholangiocarcinoma	15	IV	Primary tumor, biospy	70%	Before treatment
#2	Gallbladder cancer	31	IV	Primary tumor, biospy	90%	Before treatment
#3	Cholangiocarcinoma	14	II	Primary tumor, surgical specimen	30%	Before treatment
#4	Pancreatic cancer	28	IV	Primary tumor, cytology	70%	Before treatment
#5	Non-small cell lung cancer	20	IIIA	Primary tumor, surgical specimen	80%	Before treatment
#6	Non-small cell lung cancer	140	IIIA	Primary tumor, surgical specimen	90%	Before treatment
#7	Non-small cell lung cancer	31	IIIA	Primary tumor, surgical specimen	70%	Before treatment
#8	Non-small cell lung cancer	35	IIIB	Primary tumor, surgical specimen	90%	Before treatment
#9	Non-small cell lung cancer	35	IIB	Primary tumor, surgical specimen	70%	Before treatment
#10	Non-small cell lung cancer	25	IA	Primary tumor, surgical specimen	80%	Before treatment
#11	Non-small cell lung cancer	30	IIIA	Primary tumor, surgical specimen	90%	Before treatment
#12	Non-small cell lung cancer	35	IB	Primary tumor, surgical specimen	90%	Before treatment

Supplemental Table 2. Sequencing and coverage data

Case number	Tumor type	Sample	Mapped reads	On target	Mean depth	Uniformity	Assay
#1	Cholangiocellular carcinoma	Plasma	3,754,464	97.2%	58,575	100.0%	MB
#2	Gallbladder cancer	Plasma	2,514,168	97.1%	39,538	99.0%	MB
#3	Cholangiocellular carcinoma	Plasma	3,472,708	96.9%	53,377	98.3%	MB
#4	Pancreatic cancer	Plasma	6,094,694	96.2%	91,855	98.3%	MB
#5	Non-small cell lung cancer	Plasma	2,957,896	97.7%	54,551	96.9%	MB
#6	Non-small cell lung cancer	Plasma	1,586,321	98.7%	29,145	94.9%	MB
#7	Non-small cell lung cancer	Plasma	2,625,945	96.7%	47,506	97.1%	MB
#8	Non-small cell lung cancer	Plasma	2,476,376	96.2%	44,623	96.9%	MB
#9	Non-small cell lung cancer	Plasma	2,458,344	96.1%	43,489	93.2%	MB
#10	Non-small cell lung cancer	Plasma	867,018	97.4%	15,853	96.9%	MB
#11	Non-small cell lung cancer	Plasma	3,549,516	97.2%	64,744	96.9%	MB
#12	Non-small cell lung cancer	Plasma	2,313,442	98.8%	42,864	94.1%	MB
#1	Cholangiocellular carcinoma	Tumor	8,322,428	96.5%	128,044	98.3%	MB
#2	Gallbladder cancer	Tumor	3,735,040	98.1%	62,707	92.1%	MB
#3	Cholangiocellular carcinoma	Tumor	4,236,568	98.4%	69,233	96.9%	MB
#4	Pancreatic cancer	Tumor	5,983,945	98.7%	95,415	99.2%	MB
#5	Non-small cell lung cancer	Tumor	355,964	98.7%	6,762	96.9%	MB
#6	Non-small cell lung cancer	Tumor	470,015	98.0%	8,760	96.9%	MB
#7	Non-small cell lung cancer	Tumor	499,853	98.7%	9,401	94.9%	MB
#8	Non-small cell lung cancer	Tumor	374,209	99.1%	7,067	96.9%	MB

#9	Non-small cell lung cancer	Tumor	412,872	98.7%	7,806	96.9%	MB
#10	Non-small cell lung cancer	Tumor	401,632	97.9%	7,539	94.9%	MB
#11	Non-small cell lung cancer	Tumor	529,937	98.6%	9,896	96.9%	MB
#12	Non-small cell lung cancer	Tumor	448,788	97.9%	8,375	96.9%	MB
#1	Cholangiocellular carcinoma	Buffy	4,457,587	96.4%	1,712	93.5%	Non-MB
#2	Gallbladder cancer	Buffy	3,284,762	96.6%	1,265	88.6%	Non-MB
#3	Cholangiocellular carcinoma	Buffy	2,967,476	96.8%	1,161	87.9%	Non-MB
#4	Pancreatic cancer	Buffy	6,872,346	96.0%	2,598	95.1%	Non-MB
#5	Non-small cell lung cancer	Buffy	7,005,018	96.6%	2,598	94.1%	Non-MB
#6	Non-small cell lung cancer	Buffy	2,072,413	97.0%	731	86.6%	Non-MB
#7	Non-small cell lung cancer	Buffy	5,400,620	96.5%	2,010	96.0%	Non-MB
#8	Non-small cell lung cancer	Buffy	10,970,074	97.1%	4,068	96.1%	Non-MB
#9	Non-small cell lung cancer	Buffy	4,226,844	97.4%	1,570	96.3%	Non-MB
#10	Non-small cell lung cancer	Buffy	2,265,267	96.9%	797	88.5%	Non-MB
#11	Non-small cell lung cancer	Buffy	5,197,102	97.6%	1,959	85.2%	Non-MB
#12	Non-small cell lung cancer	Buffy	2,124,972	96.5%	744	88.6%	Non-MB
#1	Cholangiocellular carcinoma	Plasma	4,186,864	87.5%	1,322	64.6%	Non-MB
#2	Gallbladder cancer	Plasma	2,308,019	80.2%	661	76.6%	Non-MB
#3	Cholangiocellular carcinoma	Plasma	2,208,811	89.5%	735	92.3%	Non-MB
#4	Pancreatic cancer	Plasma	2,051,196	89.2%	690	88.3%	Non-MB
#5	Non-small cell lung cancer	Plasma	1,564,526	91.3%	539	93.8%	Non-MB
#6	Non-small cell lung cancer	Plasma	2,211,838	62.0%	302	87.8%	Non-MB

#7	Non-small cell lung cancer	Plasma	1,226,665	90.6%	417	91.7%	Non-MB
#8	Non-small cell lung cancer	Plasma	3,187,923	87.3%	1,026	96.6%	Non-MB
#9	Non-small cell lung cancer	Plasma	2,140,283	90.1%	704	91.7%	Non-MB
#10	Non-small cell lung cancer	Plasma	1,825,259	67.0%	336	89.0%	Non-MB
#11	Non-small cell lung cancer	Plasma	1,649,419	90.4%	560	92.6%	Non-MB
#12	Non-small cell lung cancer	Plasma	1,448,017	67.0%	257	88.0%	Non-MB
#1	Cholangiocellular carcinoma	Tumor	3,216,705	96.6%	1,147	83.0%	Non-MB
#2	Gallbladder cancer	Tumor	12,303,197	97.7%	4,634	82.4%	Non-MB
#3	Cholangiocellular carcinoma	Tumor	6,452,140	96.9%	2,454	89.7%	Non-MB
#4	Pancreatic cancer	Tumor	5,447,544	97.0%	2,086	89.0%	Non-MB
#5	Non-small cell lung cancer	Tumor	11,350,163	97.5%	4,251	81.3%	Non-MB
#6	Non-small cell lung cancer	Tumor	1,485,554	68.0%	184	75.0%	Non-MB
#7	Non-small cell lung cancer	Tumor	6,747,121	94.9%	2,440	79.6%	Non-MB
#8	Non-small cell lung cancer	Tumor	5,060,613	97.4%	1,856	95.2%	Non-MB
#9	Non-small cell lung cancer	Tumor	4,497,132	97.5%	1,655	95.7%	Non-MB
#10	Non-small cell lung cancer	Tumor	2,910,896	96.7%	980	75.3%	Non-MB
#11	Non-small cell lung cancer	Tumor	6,685,444	97.5%	2,488	81.0%	Non-MB
#12	Non-small cell lung cancer	Tumor	2,487,433	96.3%	842	77.5%	Non-MB

MB, molecular barcode; Non-MB, non-molecular barcode
